# The SWI/SNF ATP-Dependent Chromatin Remodeling Complex in Arabidopsis Responds to Environmental Changes in Temperature-Dependent Manner

**DOI:** 10.3390/ijms21030762

**Published:** 2020-01-23

**Authors:** Dominika M. Gratkowska-Zmuda, Szymon Kubala, Elzbieta Sarnowska, Pawel Cwiek, Paulina Oksinska, Jaroslaw Steciuk, Anna T. Rolicka, Magdalena Zaborowska, Ernest Bucior, Anna Maassen, Rainer Franzen, Csaba Koncz, Tomasz J. Sarnowski

**Affiliations:** 1 Institute of Biochemistry and Biophysics, Polish Academy of Sciences, 02-106 Warsaw, Poland; gratkowskad@gmail.com (D.M.G.-Z.); szymonglobus@gmail.com (S.K.); p.cwiek23@gmail.com (P.C.); p.oksinska@ibb.waw.pl (P.O.); keraj3000@gmail.com (J.S.); arolicka@gmail.com (A.T.R.); magdalena.zaborowska@ibb.waw.pl (M.Z.); ebucior@gmail.com (E.B.); maassen.anna@gmail.com (A.M.); 2 Maria Sklodowska-Curie Cancer Center Institute of Oncology, 02-781 Warsaw, Poland; 3 Faculty of Biology, University of Warsaw, 02-106 Warsaw, Poland; 4 Max-Planck Institut für Pflanzenzüchtungsforschung, Köln 50829, Germany; franzen@mpipz.mpg.de (R.F.); koncz@mpipz.mpg.de (C.K.); 5 Institute of Plant Biology, Biological Research Center of Hungarian Academy, 6726 Szeged, Hungary

**Keywords:** SWI3C, SWI/SNF, cold response, ATP-dependent chromatin remodeling, transcriptional control of gene expression

## Abstract

SWI/SNF ATP-dependent chromatin remodeling complexes (CRCs) play important roles in the regulation of transcription, cell cycle, DNA replication, repair, and hormone signaling in eukaryotes. The core of SWI/SNF CRCs composed of a SWI2/SNF2 type ATPase, a SNF5 and two of SWI3 subunits is sufficient for execution of nucleosome remodeling in vitro. The Arabidopsis genome encodes four SWI2/SNF2 ATPases, four SWI3, a single SNF5 and two SWP73 subunits. Genes of the core SWI/SNF components have critical but not fully overlapping roles during plant growth, embryogenesis, and sporophyte development. Here we show that the Arabidopsis *swi3c* mutant exhibits a phenotypic reversion when grown at lower temperature resulting in partial restoration of its embryo, root development and fertility defects. Our data indicates that the *swi3c* mutation alters the expression of several genes engaged in low temperature responses. The location of SWI3C-containing SWI/SNF CRCs on the *ICE1*, *MYB15* and *CBF1* target genes depends on the temperature conditions, and the *swi3c* mutation thus also influences the transcription of several cold-responsive (COR) genes. These findings, together with genetic analysis of *swi3c/ice1* double mutant and enhanced freezing tolerance of *swi3c* plants illustrate that SWI/SNF CRCs contribute to fine-tuning of plant growth responses to different temperature regimes.

## 1. Introduction

The SWI/SNF class of ATP-dependent chromatin remodeling complexes (CRCs), a prototype of which was first described in yeast, are conserved from fungi to plants and mammals. SWI/SNF CRCs control basic regulatory processes, such as transcription, cell cycle, replication, carcinogenesis, hormone-dependent gene expression, stress response etc. (for review see: [1]). For their full basic remodeling activity in vitro, the SWI/SNF complexes require a catalytic subunit, the Snf2-type ATPase, as well as a single SNF5 and two SWI3-type subunits, which together compose the core chromatin remodeling complex [2]. Beside core subunits, SWI/SNF CRCs carry several accessory subunits [1]. The Arabidopsis genome encodes four SWI2/SNF2-type ATPases (BRM, SYD, CHR12/MINU1, and CHR23/MINU2; [3,4,5,6]), a single SNF5-type (BSH; [7]) and four SWI3 subunits (SWI3A, B, C, D; [8]). As in mammals, the Arabidopsis core SWI/SNF subunits play important roles in the control of developmental and signaling processes [1].

SWI/SNF complexes assembled by distinct combinations of core and accessory subunits are implicated in the regulation of different cellular processes [1,8,9,10,11]. *SWI3A* and *SWI3B* are essential genes, their inactivation leads to embryo arrest at the globular stage of embryogenesis. *SWI3B* also plays an important role during gametophyte development. By contrast, *SWI3C* and *SWI3D* appear to be dispensable for embryogenesis. The *swi3c* and *swi3d* mutants are viable, although they exhibit strong developmental aberrations. The *swi3d* mutation causes a complete sterility, whereas *swi3c* mutant plants are characterized by dramatic root shortening and branching, semi-dwarfism, altered leaf and flower development, and reduced fertility [8]. However, these developmental defects are less severe when *swi3c* plants are grown at 14–16 °C compared to 20–24 °C [12]. Intriguingly, inactivation of SWP73 SWI/SNF subunit in yeast is similarly reported to cause sensitivity to elevated temperature [13]. In Arabidopsis, BRM-containing SWI/SNF CRC interacts with the histone deacetylase HD2C, which is implicated in the repression of a battery of heat-activated genes [14]. These findings suggest that the SWI/SNF CRCs are involved in temperature-dependent control of transcription but so far, the underlying molecular mechanisms are largely unclear.

Here, we show that developmental defects of the Arabidopsis *swi3c* mutant, including embryo arrest at early stages and defective root elongation, are partially reverted when plants are grown at 14 °C. Our data indicate that SWI3C-containing SWI/SNF CRCs modulate the expression of several genes involved in low temperature signaling including *ICE1, CBF1-3, MYB15, RAP2.6, ZAT10,* and *ZAT12*. SWI3C-containing SWI/SNF CRCs were found to bind to the *ICE1*, *MYB15* and *CBF1* loci and their locations on these target genes is changed by the temperature conditions. Characterization of the *swi3c/ice1* double mutant suggests a genetic interaction between *SWI3C* and *ICE1*. The *swi3c* mutation also influences the expression of downstream-acting cold-responsive (COR) genes and confers enhanced freezing tolerance. In conclusion, our data illustrate that the SWI3C-containing SWI/SNF CRCs are implicated in temperature-dependent regulation of plant growth and developmental responses.

## 2. Results

### 2.1. Lower Temperature Alleviates Phenotypic Defects Caused by Mutations of the SWI3C Core Subunit Gene of Arabidopsis SWI/SNF CRC

**Figure 1:**
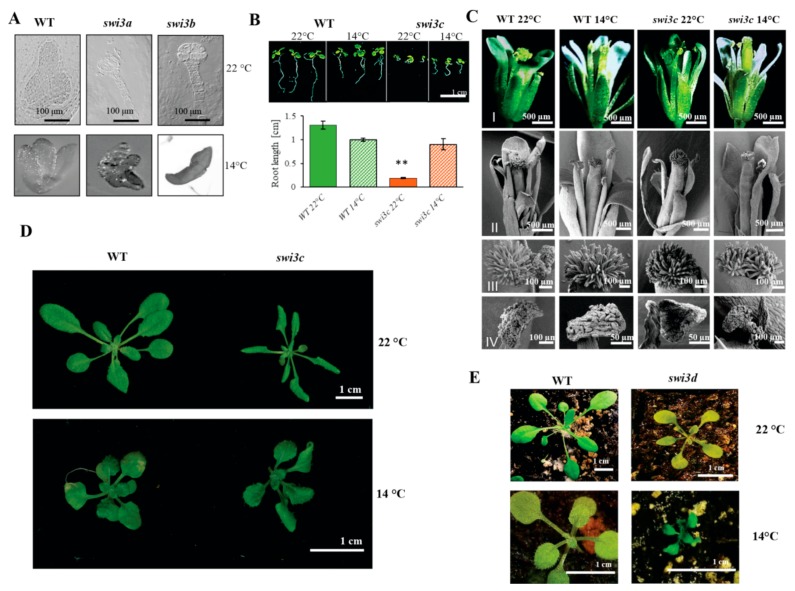
Phenotypic Characterization of Arabidopsis *swi3* Mutants Grown at 14 °C. (**A**) Nomarski image of WT, *swi3a* and *swi3b* embryos at 22°C (upper panel) and at 14°C (lower panel). (**B**) Root phenotype of 14-days-old *swi3c* line grown at 22°C and 14°C. Root length measurement showed that the *swi3c* mutant plants exhibited partial reversion of root elongation defect at 14°C. (**): Significantly different at *p* < 0.01 according to t-test, three biological replicates were used, at least ten plants from each genotype were measured. (**C**) Appearance of mature flowers (I) and analysis of their organs (II) including stigma (III) and anther (IV) by scanning microscopy in WT and *swi3c* plants grown at 22°C and 14°C. (**D**) The rosette of WT and *swi3c* plants grown at 22°C and 14°C. Scale bar 1 cm. (**E**) Comparison of 14-days-old WT and *swi3d* plants grown at 22°C and 14°C.

Inactivation of genes encoding the SWI3-type subunits of SWI/SNF CRCs results in distinct effects on Arabidopsis development. The *swi3a* and *swi3b* mutations cause lethality at the early (globular) stage of embryo development. By contrast, the *swi3c* and *swi3d* mutants are viable but exhibit severe developmental defects including reduced fertility of *swi3c* and complete sterility of *swi3d* plants grown under optimal conditions [8]. We previously observed that reducing the ambient temperature to 14–16 °C substantially improved the fertility of the *swi3c* mutant, which displayed an enhanced elongation of siliques containing viable seeds [12]. This observation has prompted us to examine how lower growth temperature affects the phenotypic traits conferred by mutations of all four Arabidopsis *SWI3* genes. When grown at 14 °C, the number of white translucent seeds carrying aborted embryos was reduced from 24.8% to 6.8% and 14.8% to 0.8% in siliques of *swi3a/+* and *swi3b/+* plants, respectively. Gametophyte lethality of *swi3b/+* line decreased from 34.5% to 11.95% as a result of decreasing the growth temperature (Table 1). Further analysis of mature seeds by PCR-based genotyping of isolated embryos confirmed that the reduction of growth temperature partially suppressed the defect of embryo development at the globular stage. In the progeny of *swi3a/+* plants, we identified mature *swi3a* embryos, although their cotyledons appeared to be underdeveloped and degenerated. Similar inspection of *swi3b/+* offspring identified *swi3b* embryos that displayed a torpedo-stage-like developmental status. Nonetheless, germination of seeds collected from *swi3a/+* or *swi3b/+* plants grown at 14 °C did not result in viable homozygous *swi3a* or *swi3b* progeny. This indicated that reduction of the growth temperature permitted the *swi3a* and *swi3b* embryos to reach later stages of development but did not completely suppress the block of embryo development by the *swi3a* or *swi3b* mutations (Figure 1A).

Decreasing the growth temperature resulted in notable alleviation of the block of root elongation (Figure 1B) and enhanced root branching [12] of the *swi3c* mutant. At 14 °C, the length of siliques and the yield of viable seeds increased considerably from 24 to 66% and 5 to 31% compared to wild type [12], respectively, although no gross changes in overall differentiation and development of flower organs and growth of *swi3c* plants were detected with the exception of slightly reduced leaf curling (Figure 1C,D, Supporting Information Figure S1). In comparison, lower growth temperature resulted in remarkable growth retardation (Figure 1E) and did not restore the fertility of *swi3d* mutant. Nonetheless, we found a nearly two-fold extension of *swi3d* silique length in plants growing at 14 °C (Supporting Information Figure S2A,B). Despite the extension of silique length, the *swi3d* mutant displayed complete sterility under both low and normal temperature conditions.

In summary, except for *swi3d* where only siliques became longer, characteristic developmental defects caused by mutations of *SWI3* core subunit genes of SWI/SNF CRCs were partially suppressed at lower temperature. As only the *swi3c* mutant produced viable homozygous progeny, further analysis of the mechanisms underlying temperature sensitivity of *swi3* mutants addressed the question how inactivation of *SWI3C* gene influences the expression of genes implicated in cold temperature signaling and downstream responses.

### 2.2. Genes Involved in Low Temperature Responses Show Altered Expression in the Swi3c Mutant

**Figure 2:**
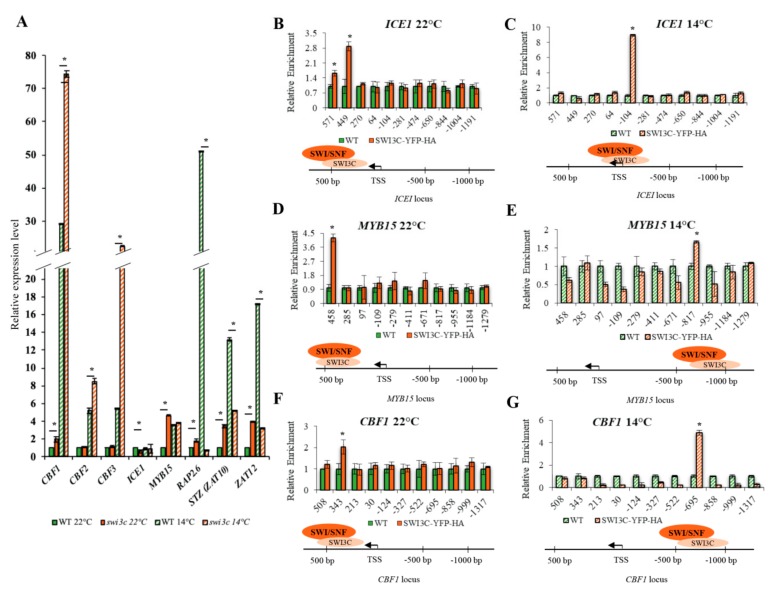
Altered Transcription of Genes Involved in Cold Signaling in the *swi3c* Mutant. (**A**) Relative expression levels of genes of cold signaling pathway in wild type and *swi3c* plants grown at 22°C and 14° C. Plants were in a developmental stage with 12 rosette leaves > 1mm length according to Boyes et al. (2001) [24]. Asterisks indicate significant difference (*): *p*< 0.05 according to t-test. (**B**) SWI3C binding to 5′-UTR region of ICE1 at 22°C and (**C**) at 14°C. (**D**) SWI3C binding to the promoter region of MYB15 at 22 °C and (**E**) at 14°C. (**F**) Peak position of SWI3C in the CBF1 promoter at 22°C and (**G**) at 14 °C. The TA3 transposon served as reference gene. Three biological and three technical replicates were used. Plants were in the principal stage with 12 rosette leaves > 1mm length according to Boyes et al. (2001) [24]. Asterisks indicate significant difference from wild type (*): *p* < 0.05 according to t-test. Bars refer to SD.

Transcription responses to cold temperature are controlled by the ICE1 (Inducer of CBF Expression 1) Myc transcription factor (TF), which stimulates the expression of three C-Repeat Binding Factors (CBFs) of APETALA2/ETHYLENE RESPONSE FACTOR TF family. The function of ICE1 master regulator of CBFs is regulated post-translationally [15,16,17]. The HOS1 ubiquitin ligase targets ICE1 for proteasomal degradation, which is counteracted by SIZ1-mediated sumoylation of ICE1 [18]. CBFs are central activators of cold-responsive (COR) genes, which are either positively or negatively regulated by the CBF-controlled TFs RAP2.1, RAP2.6 and RAP2.7 [19]. CBF expression is repressed by MYB15, which is inactivated by ICE1 under cold stress, as well as by the C2H2 zinc finger TF ZAT12 [20,21]. Another zinc finger TF ZAT10 appears to be activated by CBF3 and acts as a repressor of a set of *COR* genes [22]. Quantitative real-time PCR measurement indicated that the *ICE1* transcript levels were comparable in wild type and *swi3c* mutant plants at 14 °C, although *ICE1* mRNA levels were 2-fold lower at 22 °C in the *swi3c* mutant (Figure 2A). Similarly, *HOS1* and *SIZ1* mRNA levels were similar in wild type and *swi3c* plants under both temperature conditions. Although *MYB15* showed 4.5-fold higher expression in *swi3c* plants under normal growth condition, its mRNA level did not differ from wild type at 14°C. In comparison, expression levels of *RAP2.6*, *ZAT10* and *ZAT12* were elevated (respectively 2, 4 and 4.5-fold) at 22 °C but showed a notable down-regulation (50, 3 and 6.5-fold, respectively) at 14 °C in *swi3c* compared to wild type plants. Transcript levels of *CBF2* and *CBF3* did not differ at normal temperature, but *CBF1* expression was somewhat (2-fold) higher in *swi3c* plants as in wild type. However, at 14 °C all three *CBF* genes showed higher (2.5, 1.5 and 4.0-fold, respectively) induction in the *swi3c* mutant compared to wild type. In conclusion, this data indicated that the *swi3c* mutation elevates the steady-state levels of *CBF* mRNAs, while decreases in parallel the transcript levels of ZAT12 CBF repressor and downstream-acting ZAT10 and RAP2.6 negative regulators of subsets of *COR* genes at 14 °C. In addition, differential up-regulation of *MYB15*, *RAP2.6*, *ZAT10* and *ZAT12* expression levels at 22 °C in the *swi3c* mutant suggested that SWI3C-containing SWI/SNF CRCs could play a role in temperature-dependent modulation of transcription responses.

### 2.3. Temperature-Dependent Shift of Localization of SWI3C-Containing SWI/SNF CRCs in the 5′-UTR Regions of ICE1, MYB15 and CBF1 Genes

To map chromatin localization of SWI3C-containing SWI/SNF CRCs on genes involved in cold signaling, the *swi3c* mutant was complemented by a SWI3C-YFP-HA gene construct carried by the Agrobacterium binary vector pEarley101 [23]. Constitutive expression of SWI3C-YFP-HA restored at both 22 °C and 14 °C the phenotypic defects, such as leaf curling (Supporting Information Figure S3A), block of root elongation and sterility of the *swi3c* mutant to wild type and resulted in the production of intact SWI3C-YFP-HA protein of 115 kDa detected by western blotting with anti-GFP antibody (Supporting Informationn Figure S3B). Chromatin immunoprecipitation followed by quantitative PCR (ChIP-qPCR) was performed with nuclear extracts prepared from formaldehyde cross-linked *swi3c/*SWI3C-YFP-HA and control wild type plants using primers for amplification of 120–160 bp segments of 5′-UTRs located between positions +450 and −1200 relative to the transcription start site (TSS). In the promoter region of *ICE1* gene, SWI3C-YFP-HA was localized between positions +449 and +571 at 22 °C (Figure 2B). The peak value of SWI3C-YFP-HA increased approximately 3-fold and its localization was shifted to position −104 at 14 °C (Figure 2C). In the 5′-UTR of *MYB15*, SWI3C-YFP-HA was localized around position of +458 at 22°C (Figure 2D) but at 14 °C the level of SWI3C-YFP-HA decreased approximately 2-fold and its localization was shifted to position −817 (Figure 2E). A similar change in positioning of SWI3C-YFP-HA was detected in the 5′-UTR region of the *CBF1* gene, in which the peak of ChIP-signal was detected around position +343 at 22 °C (Figure 2F), while at 14 °C the localization was shifted to position −695 in parallel with approximately 2.5 fold increase of signal intensity (Figure 2G). The ChIP-PCR data thus indicated that in the case of *ICE1*, lowering the temperature resulted in a shift toward proximal position of TSS, whereas in the 5′-UTR of *MYB15* the localization of SWI3C-YFP-HA was changed to a more distant upstream position. On the other hand, the localization of SWI3C-YFP-HA both downstream and upstream of TSS of the *CBF1* gene suggested that SWI3C-containing SWI/SNF CRCs might act as negative regulators because *CBF1* transcript levels were 2 and 2.5-fold higher in the *swi3c* mutant compared to wild type. Finally, localization of SWI3C-YFP-HA closer to TSS did not seem to affect *ICE1* transcription, which is comparable between wild type and the *swi3c* mutant at 14 °C. Nevertheless, more upstream localization of SWI3C correlated with a 2-fold reduction of *ICE1* mRNA levels at 22 °C. In summary, these data indicated that the localization of SWI3C-SWI/SNF CRCs is dynamically changed in the promoter regions of examined genes in a temperature-dependent manner.

Analysis of predicted transcription factor binding sites in the SWI3C-binding region using the MEME software [25] identified several potential *cis-*regulatory elements in the SWI3C-targeted regions of *MYB15*, *ICE1* and *CBF1* genes (Supporting Information Table S2). However, subsequent GOMo analysis did not confirm potential biological roles of motives derived from the MEME analyses (Supporting Information Table S2). Additional predictions obtained by using the AGRIS server (AGRIS; http://arabidopsis.med.ohio-state.edu/) suggested potential conservation of a MYB4 binding site (ACCTAAC, [26] at mapped positions of SWI3C binding to *CBF1* locus at 22 °C. In addition, a SWI3C-binding domain mapped around position −695 relative to the TSS of *CBF1* at 14 °C was predicted to carry RAV1-A (CAACA), SORLIP2 (GGGCC) and LFY (LEAFY) binding (CCAGTG) motives. The RAV1-A binding site is recognized by the ABI3/VP1 transcription factors family [27], the SORLIP2 motif is involved in the control of light-regulated gene expression [28], whereas the LFY consensus is recognized by the LEAFY transcription factor, which is reported to mediate the recruitment of SWI/SNF CRCs to their target site on e.g., *AP3* (*APETALA 3*) promoter [29]. The 5′-UTRs of *ICE1* and *MYB15* genes however lacked these motives. The only known predicted TF-binding site in these 5′-UTRs was a BELLRINGER motif (AAATTAAA), which was identified in the SWI3C-binding region in *MYB15* (position −817) at 14 °C. This motif is used by homeobox/MYB transcription factors of regulatory networks controlling development, metabolism and responses to biotic and abiotic stresses in Arabidopsis [30] (Supporting Information Table S2).

### 2.4. The Swi3c Mutation Changes Nucleosome Positioning on the ICE1, MYB15 and CBF1 Loci

**Figure 3:**
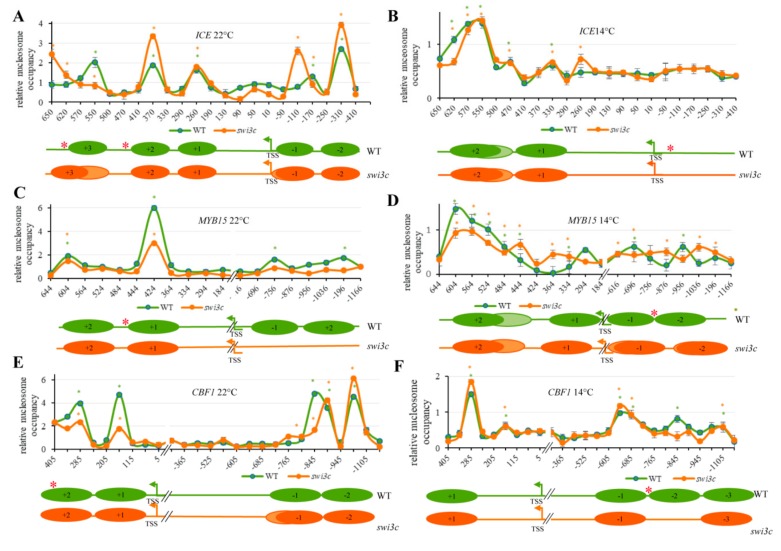
The Effect of SWI3C Inactivation on Nucleosomal Structures of *ICE1*, *MYB15* and *CBF1* Genes. (**A**) Relative nucleosomes occupancy on the *ICE1* locus in WT and *swi3c* plants grown at 22°C and (**B**) 14°C. (**C**) Relative nucleosome occupancy on *MYB15* in WT and *swi3c* plants at 22°C and (**D**) 14°C. (**E**) Relative nucleosome occupancy on *CBF1* in WT and *swi3c* plants at 22°C and (**F**) 14°C. Plants were in the principal stage with 12 rosette leaves > 1mm length according to Boyes et al. (2001) [24]. Three biological and three technical replicates were used. The fraction of undigested genomic DNA amplified for each amplicon was normalized to that of the −73 position of GYPSY-LIKE retrotransposon (At4g07700) as control. Lower panels in each figure section show schematic illustration of nucleosome positioning and dynamics. Red asterisks indicate the localization of SWI3C protein determined by ChIP-qPCR analysis. Dark-color circles indicate positioned nucleosomes, and light colors mark non-positioned nucleosomes. Green and orange asterisks (*) indicate significant (*p* < 0.05) protection of DNA from MNase digestion in WT or *swi3c*, respectively.

To correlate the SWI3C chromatin localization data with alterations of nucleosome positioning in the *swi3c* mutant, micrococcal nuclease protection qPCR (MNase-qPCR) assays were performed using a set of primers positioned between +600 and −1100 relatively to TSSs of the *ICE1*, *MYB15* and *CBF1* genes. In wild type plants grown at 22 °C five nucleosomes protected from MNase digestion were mapped to positions −200 (−1 nucleosome), −360 (−2 nucleosome), +225 bp (+1), + 360 bp (+2) and +530 (+3) relative to the TSS of *ICE1*. In the *swi3c* mutant the TSS proximal nucleosome exhibited a slight shift to position −140 and fuzziness, as well as increased occupancy, correlating with lower than wild type transcript levels at 22°C. In addition, the *swi3c* mutation resulted in enhanced positioning of nucleosome +2 and fuzziness of nucleosome +3, both located close to mapped position of SWI3C at normal temperature (Figure 3A). By contrast, wild type and *swi3c* plants grown under decreased temperature did not show a change in nucleosome occupancy correlating with no difference in *ICE1* transcription between wild type and *swi3c* plants under this condition (Figure 3B).

In case of the *MYB15* locus four nucleosomes protected from MNase digestion were mapped in wild type plants grown at 22 °C. Two of these were located in the distal promoter region centered to positions −816 (−1 nucleosome) and −1130 (−2 nucleosome), whereas the other two nucleosomes were mapped into the gene body around positions + 394 bp (+1) and +574 (+2). In correlation with a 4.5-fold increase of *MYB15* transcript levels at 22°C, in the *swi3c* mutant we found a complete loss of nucleosomes −1 and −2, as well as decreased occupancy of nucleosome +1 located in the vicinity of mapped SWI3C-binding region at position +458 (Figure 3C). At 14 °C, the positioning of nucleosomes was slightly shifted to positions −726 bp (−1) 1000 (−2), +244 (+1) and 544 (+2). Compared to wild type, in the *swi3c* mutant the positioning of nucleosomes was fuzzier, and nucleosomes +1 and −2 were slightly shifted (Figure 3D). However, no striking difference was found in positioning of nucleosomes correlating with no difference in *MYB15* mRNA levels between wild type and *swi3c* plants at 14 °C.

In both wild type and *swi3c* plants grown under normal temperature four nucleosomes were protected from MNase in the *CBF1* locus. Two nucleosomes were located in the distal promoter region centered to positions −815 (−1) and −990 (−2), while others were situated at positions the +120 (+1) and + 325 (+2) in the gene body. In correlation with a 2-fold increase of *CBF1* mRNA levels compared to wild type at 22 °C, the −1 nucleosome exhibited a slight fuzziness, and nucleosomes +1 and +2 in the vicinity of identified SWI3C binding site at position +343 (Figure 3E) showed decreased occupancy in the *swi3c* mutant (Figure 3F). At 14°C, the positions of nucleosomes were shifted to positions −680 (−1), −855 bp (−2) −1000 (−3) and +270. In contrast to normal temperature, at 14 °C the positioning of nucleosome +1 did not change. However, a complete loss of nucleosome −2 was detected close to the location of mapped SWI3C target region at position −695. This observation, together with a 2.5-fold increase of *CBF1* mRNA levels in *swi3c* plants indicated that lack of SWI3C caused two different, temperature-dependent changes to generate more open chromatin structure in the promoter region of *CBF1*gene (Figure 3F).

As this analysis did not show a clear correlation between nucleosome positioning, SWI3C occupancy and *ICE1, MYB15* and *CBF1* gene expression, we reanalyzed the data and compared nucleosome positioning and transcript level detected in the WT 22 °C *vs* WT 14°C and *swi3c* 22 °C *vs swi3c* 14 °C datasets (Supporting Information Figure S4A,B). This indicated that both WT and *swi3c* mutant plants responded to decreased temperature in a similar way concerning *ICE1* and *CBF1* genes (Supporting Information Figure S4B), although some nucleosome positioning changed in temperature-dependent manner (Supporting Information Figure S4A). The nucleosome positions differed between WT and *swi3c* mutant at 22°C and *CBF1* expression in the mutant plants was much higher than in WT in both temperature conditions (Figure 2A). Interestingly, the highest changes in transcription for *swi3c* mutant *vs* WT at 22 °C were observed for *MYB15* gene (Figure 2A). In the *swi3c* mutant, the *MYB15* gene responded to decreased growth temperature in different way than in WT, where *MYB15* expression was elevated at 14°C, whereas *swi3c* plants exhibited similar levels of *MYB15* mRNA regardless of temperature conditions (Supporting Information Figure S4B). Moreover, we found that the nucleosomes −1 and −2 at 22 °C on *MYB15* in the in *swi3c* mutant were missing from promoter region close to TSS, which could explain the almost 5-fold highest expression at 22°C compared to WT. At lower temperature, the positioning of nucleosomes in this region is observed and the activation of transcription and proper response (elevated expression) occurred in WT plants. By contrast, the *MYB15* expression level in the *swi3c* mutant exposed to lower temperature resembled WT under the same conditions. Thus, although in the *MYB15* promoter region two nucleosomes were located in cold, the *swi3c* mutation did not seem to alter *MYB15* transcription in response to low temperature.

In conclusion, the MNase nucleosome mapping data only partially supported the results of transcript measurements and SWI3C ChIP-qRT-PCR assays revealing that the lack of SWI3C subunit of SWI/SNF CRCs results in distinct temperature-dependent changes in the chromatin structure of genes involved in cold signaling. This suggests that likely other remodelers (i.e., other classes of SWI/SNF complexes) are also involved in transcriptional control of genes in the cold response pathway in response to decreased temperature. However, replacement of the missing SWI3C-SWI/SNF complex by alternative CRCs remains to be confirmed by further studies.

### 2.5. Genetic Interactions Between SWI3C and ICE1

*ICE1* is a key positive regulator of the cold signaling pathway. To test how inactivation of *ICE1* affect the cold temperature-dependent suppression of *swi3c* developmental and fertility defects, the *swi3c-1* mutation [8] was introduced by crossing into the *ice1* (SALK_068119) T-DNA insertion mutant. In the SALK_068119 mutant a T-DNA insertion in exon 4 of *ICE1* resulted in a C-terminal truncation of the coding domain, which showed still active transcription 5′ upstream of the insertion site (Supporting Information Figure S5A,B). Nonetheless, the mutation appeared to cause at least a partial loss of ICE1 function because in the F2 offspring of *swi3c/+ice1/+* hybrid we failed to identify homozygous *swi3c ice1* double mutants. In the siliques of *swi3c/+ice1* F2 progeny we did not observe embryo lethality, which suggested that *swi3 ice1* homozygous seeds were either not viable or incapable of germinating in soil under normal conditions. When germinating 216 F2 seed of a *swi3c/+ice1* plant on 0.5 MS medium only 8 (3.7%) *swi3 ice1* seedlings were identified by PCR-based genotyping indicating a significant deviation from the expected 3:1 ratio. Upon transfer into soil, the double mutant plants displayed an enhanced severity of *swi3c* developmental defects, including enhanced dwarfism and reduction of leaf blade size (Supporting Information Figure S6A) and complete sterility. Comparative microscopic analysis of developmental status of embryos using Nomarski optics indicated retarded development of embryos in both *swi3c* and *ice1* mutants compared to wild type plants. However, both *swi3c* and *ice1* single mutants could produce viable progeny, whereas in the double *swi3c ice1* mutant pollen tubes could not germinate into the embryo sac and fertilization did not occur resulting in a failure of embryo formation (Supporting Information Figure S6B).

### 2.6. Inactivation of SWI3C Alters the Expression of Cold-Responsive (COR) Genes

The expression of cold-responsive maker genes *ADH1* (Alcohol Dehydrogenase 1), *COR15A* (Cold-Regulated 15A), *COR47* (Cold-Regulated 47), *KIN1* (Kinase 1), *KIN2* (Kinase 2), *RD22* (Responsive to Desiccation 22), and *RD29A* (Responsive to Desiccation 29A) [19,31,32] in the *swi3c* mutant was compared to wild type by quantitative real-time (qRT-PCR). Under normal conditions (22 °C), 4.5-fold elevated expression of *MYB15* (Figure 2A) correlated with 2 to 4-fold reduced expression of *COR15A*, *COR47* and *RD29A* genes in the *swi3c* mutants compared to wild type plants (Supporting Information Figure S6C). By contrast in *swi3c* plants growing at 14 °C we detected 2.0-fold and 1.5-fold lower expression of *ADH1* and *COR15A* genes compared to WT.. We also found a discrete up-regulation of *COR47* and 2.0-fold elevated expression of *RD22* and *RD29A* genes in the *swi3c* mutant compared to WT plants grown at 14 °C. In summary, these results indicated an involvement of SWI3C in transcriptional regulation of *COR* genes under both normal and low temperature conditions.

### 2.7. Swi3c Mutant Exhibits Enhanced Freezing Tolerance

**Figure 4:**
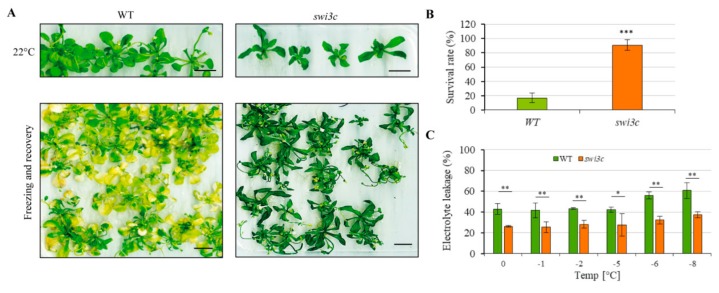
The *swi3c* Mutant Exhibits Enhanced Freezing Tolerance. (**A**) *swi3c* plants are more tolerant to freezing than wild type control. Upper panel: 3-weeks old WT and *swi3c* plants grown on plates under long day conditions. Lower panel: WT and *swi3c* plants exposed to freezing and subsequently recovered for seven days under long day conditions at 22°C. (**B**) The survival rate of wild type and *swi3c* plants subjected to freezing tolerance assay indicate enhanced freezing tolerance of *swi3c* plants. (**C**) *swi3c* plants exhibit lower electrolyte leakage after freezing damage than WT plants further confirming their enhanced freezing tolerance. Asterisks indicate significant difference from wild type (*): *p < 0.05*; (**): *p < 0.01* (***): *p <0.001* according to t-test. Error bars refer to SD.

Overexpression of CBFs in cooperation with other cold-regulated first-wave transcription factors was demonstrated to increase freezing tolerance [33]. Therefore, we examined the freezing tolerance trait of *swi3c* mutant by performing a freezing-survival assays. Wild type and *swi3c* plants were moved to 4 °C, and then within 30 min the temperature decreased to 0 °C. Subsequently, the temperature decreased by 1°C every hour until it reached −8 °C. Next, the plants were moved to a growth chamber with long day and 22 °C conditions. After 7 days recovery period, the number of surviving plants was determined. We found that the survival rate for wild type plants was approximately 20% while for *swi3c* it reached up to −90% (Figure 4A,B) indicating enhanced freezing tolerance. We also found that the constitutive expression of SWI3C-YFP-HA restored, upon freezing, the phenotypic defects exhibited by the *swi3c* plants (Supporting Information Figure S7). To further confirm enhanced freezing tolerance of *swi3c* mutant, we performed electrolyte leakage assays [34] (and determined the EL50 value (the temperature at which 50% of cellular electrolytes was released due to freezing tolerance). For wild type plants, the EL50 value was ~ −6 °C, whereas the *swi3c* mutant exhibited only ~ 30% of cellular electrolyte release at the same temperature (Figure 4C). Collectively, these results demonstrated that the *swi3c* mutant exhibited increased freezing tolerance.

## 3. Discussion

Plants, as sessile organisms, are constantly exposed to various abiotic and biotic stresses. To be able to survive in a changing environment, plants evolved numerous defense mechanisms allowing them to survive under stress conditions. The phenotypic plasticity plays a crucial role in adaptation to changing environmental conditions, thanks to which plants developed adaptive defense reactions to response to unfavorable conditions. Epigenetic changes occurring at the chromatin level play a pivotal role in the genotype-environment interaction [35].

Exposure to low temperature stimulates signaling pathways that control the expression of genes conferring plant tolerance to cold stress [36]. *ICE1* (*Inducer of CBF Expression 1*), also called SCREAM1 [37], is a key positive regulator of the cold-responsive pathway, which is activated by sumoylation. ICE1 is critical for induction of the *CBF* (*C-Repeat Binding Factor*) regulon and inhibition of the *CBF* repressor MYB15 [20]. ICE1 is negatively regulated through ubiquitination by the HOS1 (High Expression of Osmotically Responsive Genes 1) RING finger-type ubiquitin E3 ligase, which stimulates its proteasomal degradation [18]. CBFs cross-regulate their own transcription [22] and bind to *cis*-elements in the promoters of *COR* genes and activate their transcription [38]. The ICE1-MYB15-CBF signaling module plays a major role in adaptation and acclimation of plants to cold stress [39].

Adaptation to abiotic stresses is accompanied by either activation or inactivation of a large number of genes, implying changes in their chromatin structures. Alterations in the chromatin structure are induced by DNA methylation, modification of core histone tails, exchange of histone variants, and altered positioning of nucleosomes [40,41]. Histone deacetylase HD2C was reported recently to play an important role in plant adaptation to temperature changes by modulating the transcription of cold-responsive genes *COR15A* and *COR47* [42]. HD2C is known to co-operate with SWI/SNF chromatin remodeling complexes in response to high temperature in Arabidopsis [14]; however, it is yet unknown whether HD2C interaction with SWI/SNF CRCs plays also a role in plant responses to cold temperature, which is suggested by the observation that reduced fertility of plants carrying mutations of the SWI3C core subunit of SWI/SNF CRCs is largely alleviated by decreasing the growth temperature to 14°C [12].

In this report, we investigated cold temperature sensitivity of developmental defects caused by mutations of SWI3 core subunit genes of Arabidopsis SWI/SNF CRCs. We showed that decreasing the growth temperature to 14°C permitted the *swi3a* and *swi3b* mutant embryos to develop close to final maturation, although their development was arrested at the globular stage at 22 °C. Similarly, the *SWI3B*-dependent female gametophytic defect is partially restored by lowering the growth temperature to 14 °C. In case of the *swi3c* mutant, in addition to improved fertility, lowering the temperature partially suppressed the block of root elongation and enhanced root branching, and leaf curling. As exception, developmental defects of the *swi3d* mutant were not restored by decreased growth temperature. The plants growing at decreased temperature exhibited even more retarded growth but displayed a slight extension of siliques compared to *swi3d* plants grown at 22 °C. In contrast to the *swi3c* mutant, *swi3d* plants did not improve their fertility at 14°C and remained fully sterile. The differential behavior of *swi3c* and *swi3d* mutants further supports our previous findings that SWI3C and SWI3D are present in different classes of SWI/SNF CRCs [8] and suggests that SWI3D-containing SWI/SNF CRCs class acts distinctly from temperature signaling pathways.

Inactivation of SWI3C was found to alter transcriptional regulation of several genes acting in the cold signaling pathway, including *CBF1-3*, *ICE1, MYB15, RAP2.6, ZAT10* and *ZAT12*. Interestingly, the transcription of these genes was differentially deregulated in the *swi3c* mutant at 22 and 14 °C. This suggested that the function of SWI3C-containing SWI/SNF CRCs is differently regulated depending on the ambient temperature. The *swi3c* mutation was found to enhance transcription of the *CBF1-3* genes at 14°C, which normally correlates with enhanced cold tolerance [43]. Subsequent chromatin immunoprecipitation study indicated direct targeting of SWI3C to the *ICE1, MYB15* and *CBF1* loci, and in all three cases SWI3C was localized at 22°C 3′ downstream of TSS in these genes. Although unexpected, this observation is in line with recent mapping studies of human [44] and Arabidopsis [45] SWI/SNF CRCs, which were located in various gene regions. Similarly, SWI/SNF CRCs in Drosophila were observed to bind gene body regions affecting transcription elongation [46] and RNA polymerase II pausing [47]. The location of SWI3C on the *ICE1, CBF1* and *MYB15* loci has remarkably changed in a temperature-dependent manner and was shifted 5′-upstream of TSSs in the promoter regions of *ICE1*, *MYB15* and *CBF1* genes.

In the case of the *CBF1* gene, SWI3C-binding under control conditions occurred in close proximity to a MYB4-binding motif, which is targeted by various MYB transcription factors including MYB15 [20], another regulatory target of SWI3C-SWI/SNF CRCs. By contrast, under decreased temperature SWI3C shifted from this site in the gene body to the putative RAV1-A, SORLIP2 and LFY (LEAFY) consensus binding sites in the distal *CBF1* promoter region. While the LEAFY transcription factor is a known binding partner of SWI/SNF CRCs, there is so far no report on the regulatory interdependence of RAV1 and SWI/SNF. The SORLIP2 motif was identified in the promoter regions of phytochrome A-regulated genes [28] and the BRM ATPase subunit of SWI/SNF CRCs is reported to interact directly with PIF1 (PHYTOCHROME-INTERACTING-PARTNER 1) [48]. Nonetheless, so far, no regulatory link was identified between SORLIP2 and SWI/SNF. A different localization of SWI3C on the *CBF1* gene indicated two different temperature-dependent modes of transcriptional regulation by SWI/SNF-mediated chromatin remodeling at 22 and 14°C. By contrast, different localization of SWI3C- SWI/SNF CRCs on the *ICE1* locus, which is not coupled to altered transcription at 14°C, might reflect altered chromatin loop formation, transcription factor binding, histone modifications, etc. [47].

On the *CBF1*, *ICE1,* and *MYB15* loci the chromatin nucleosome structure varies in and between wild type and *swi3c* mutant plants depending on the growth temperature. Lower transcription of *ICE1* in the *swi3c* mutant at 22°C correlates with altered (enhanced) positioning and shift of nucleosomes. An opposite effect is found on the *MYB15* locus, where we observed an absence (promoter region) and decreased positioning (gene body) of particular nucleosomes. Decreased positioning of nucleosomes in the gene body and slight fuzziness of nucleosomes in the promoter region correlate with enhanced transcription of *CBF1* and *MYB15* genes under normal growth condition in the *swi3c* mutant. These findings are in line with the results showing that the presence of SWI/SNF CRCs in the gene body plays an important role in the control of nucleosome density during transcriptional elongation in Drosophila [46]. The contrasting situation was observed under a lower growth temperature where the chromatin structure on *ICE1* did not differ from the wild type, whereas only some fuzziness of nucleosomes was detected on *MYB15*, while the expression of these genes was not altered in the *swi3c* mutant compared to wild type. These results suggest that inactivation of SWI3C has no effect on the *MYB15* gene expression at decreased temperature. However, the comparative analysis of *MYB15* expression levels in WT and *swi3c* plants growing at 22°C vs. 14°C indicated that indeed WT and *swi3c* exhibit substantially different activation modes of the *MYB15* gene. In WT plants growing at decreased temperature, *MYB15* activation occurs but the *swi3c* plants are unable to elevate *MYB15* expression under these conditions. By contrast, the *swi3c* mutant was still able to elevate *CBF1* expression under decreased temperature condition compared to *swi3c* plants grown at 22°C, although under both temperature conditions the expression of *MYB15* was higher in *swi3c* than in WT plants. Interestingly, on the *CBF1* gene, a loss of nucleosome-2 was observed at 14°C, corresponding to the position of potential *cis*-regulatory sequences and enhanced transcription of *CBF1* in the *swi3c* mutant. Collectively, these findings indicate that SWI/SNF CRCs are likely implicated in chromatin remodeling of *ICE1, MYB15* and *CBF1* transcription units under changing ambient temperature.

**Figure 5:**
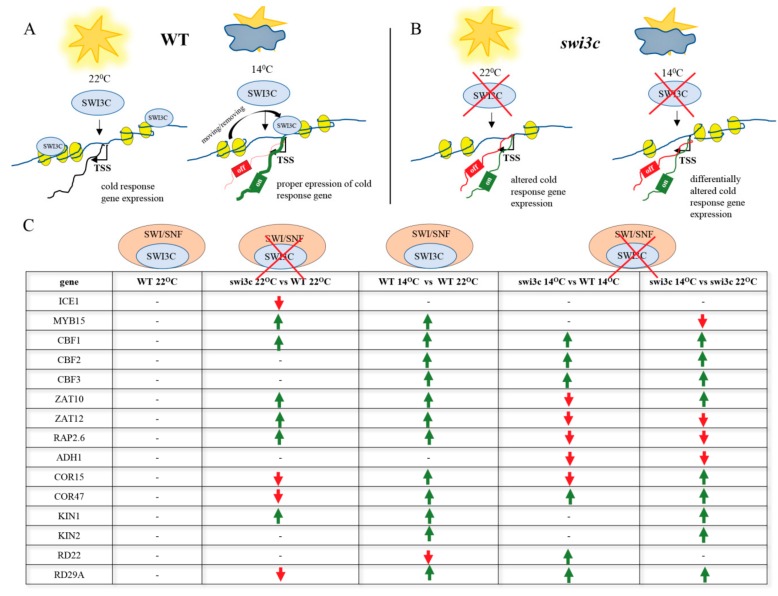
The Action of SWI3C-containing SWI/SNF CRCs in the Transcriptional Control of Genes in the Cold-Responsive Pathway Depends on the Growth Temperature. (**A**) Schematic model illustrating the role of intact SWI3C-containing SWI/SNF CRCs in the transcriptional control of *ICE1*, *CBF1* and *MYB15* genes in WT plants. The SWI3C-containing SWI/SNF complex modulates the expression of cold-responsive genes in wild type plants at 22°C in different manner than during growth at 14°C. The position of SWI3C-SWI/SNF CRC localization differs depending on the temperature. Blue circles indicate SWI3C protein and yellow ellipses represent nucleosomes. (**B**) Schematic model illustrating the impact of SWI3C inactivation on transcription of *ICE1*, *CBF1* and *MYB15* genes in *swi3c* plants. Blue circles symbolize the SWI3C protein and yellow ellipses represent nucleosomes.(**C**) The overview of transcriptional changes of cold-responsive genes in wild type and *swi3c* mutant plants. Green arrows mark elevated expression, red arrows indicate decreased expression.

The analysis of *swi3c/ice1* double mutant shows that simultaneous inactivation of *ICE1* and *SWI3C* has a dramatic effect on Arabidopsis development, i.e., the plants were severely dwarfed and completely sterile, while both *swi3c* and *ice1* single mutants produced some seeds [8,49]. Consistently, further investigation of expression of cold-responsive genes indicated their temperature-dependent deregulation in the *swi3c* mutant plants. These results suggest that *SWI3C* is involved in fine-tuning of cold responses in Arabidopsis (Figure 5A–C), in addition to its previously documented roles in modulation of leaf and flower development and hormone signaling pathways [1,11,12]. This conclusion is supported by the fact that *swi3c* plants exhibit an enhanced freezing tolerance, which was demonstrated by their increased survival rate and decreased electrolyte leakage at −8°C. It was recently shown that overexpression of CBFs increases freezing tolerance in Arabidopsis [33]. Thus, enhanced freezing tolerance of the *swi3c* mutant is consistent with observed overexpression of *CBF1-3* genes under decreased temperature.

## 4. Materials and Methods

### 4.1. Plant Lines and Growth Conditions

Wild type and mutant lines were of the *Arabidopsis thaliana L. Heynh.* Columbia-0 (Col-0 further referred as WT) ecotype (Lehleseeds, Round Rock, TX, USA). The *swi3a-1*, *swi3b-1, swi3c-1* and *swi3d-1* mutant alleles (referred further as *swi3a*, *swi3b*, *swi3c* and *swi3d*, respectively) were characterized previously [8]. The *ice1* (SALK_068119) T-DNA insertion mutant was obtained from the Nottingham Arabidopsis Stock Centre [50]. PCR primers used for genotyping are listed in Supporting Information Table S1.

Seeds were sown on soil or plated on half-strength MS medium (Sigma-Aldrich) containing 0.5% sucrose and 0.8% agar (pH 5.8). Plants were grown under long day (LD) conditions (16h light/8h dark) at 22°C or 14°C, 70% humidity and 140 mM m^–2^ s^–1^ light intensity. For qRT-PCR, ChIP-qPCR and MNase-qPCR analyses plant material was collected six hours after the onset of the light period from the aerial part of plants in principal stage growth with 12 rosette leaves > 1mm length according to Boyes et al. (2001) [24]. For each experiment, three biological and three technical replicates were used. For the assessment of phenotypic alterations three biological replicates were used.

### 4.2. Construction of Genetically Complemented Plants by Expression of YFP-HA Tagged SWI3C Protein

The *SWI3C* cDNA was cloned into pDONR207, sequenced, and moved into pEarley101 [23] using the Gateway procedure. Transgenic lines were generated by floral dip transformation of *swi3c-1/+* plants using the Agrobacterium GV3101 (pMP90) strain [51,52]. Primary transformants were selected based on their BASTA (100µM) resistance. Homozygous *swi3c* plants complemented with the SWI3C-YFP-HA construct were identified by PCR-based genotyping and subjected to subsequent analysis of their phenotypic traits. The expression of SWI3C-YFP-HA protein in *swi3c/*SWI3C-YFP-HA plants was confirmed by Western blotting using a rat anti-GFP antibody (Chromotek).

### 4.3. RNA Extraction and qRT-PCR Analysis

RNA was extracted using RNeasy plant kit (Qiagen). Traces of genomic DNA were removed using a TURBO DNA-free kit (Ambion). 2.5 μg of total RNA was reverse-transcribed using a first-strand cDNA synthesis kit (Roche). qRT-PCR assays were performed with SYBR Green Master mix (BioRad) and specific primers for PCR amplification. qRT-PCR data were recorded and analyzed using iQ-PCR (BioRad) or LightCycler480 (Roche) equipment and software according to manufacturers’ recommendations. *PP2A* and *UBQ5* (AT1G13320 and AT3G62250, respectively) mRNAs were used as reference. The relative transcript level of each gene was determined by the 2^-ΔΔCt^ method [53]. For each primer pair, the primer efficiency was measured and melting curve analyzed. For each experiment, three biological and three technical replicates were used. Primers used for qRT-PCR study are listed in Supporting Information Table S1.

### 4.4. Chromatin Immunoprecipitation (ChIP)

Chromatin was isolated from 2g samples of WT and *swi3c/*SWI3C-YFP-HA seedlings in the principal growth stage 1.12 [24] grown at 22ºC or 14ºC, respectively, and sonicated according to [54] with the exception that instead of pepstatin A and aprotinin, 1% PIC (Sigma), 1mM PMSF and 5mM β-me were included in the extraction buffer. Cross-linked SWI3C-YFP-HA samples were subjected to immunoprecipitation using 25μL of GFP-TRAP beads (Chromotek) according to the manufacturer’s protocol. The percentage of input was calculated using 2^-ΔΔCt^ method [53]. To facilitate the comparison of different samples, the calculated percent input of wild type was set to 1. The relative enrichment represents the fold change compared to the wild type. The exon region of *TA3* was used as negative control [55]. For each experiment three biological and three technical replicates were used.

### 4.5. Mapping of Nucleosome Locations Using MNase Protection Assay

For the mapping of nucleosome positions, 2g samples of WT and *swi3c* seedlings in the principal growth stage 1.12 [56] grown at 22ºC or 14ºC, respectively, were proceeded according to Saleh et al., (2008) [54] and Zhu et al., (2013) [57] with minor modifications (i.e., the buffer contained 1% proteinase inhibitor cocktail (Sigma) instead of pepstatin A and aprotinin). 200 Kunitz units of Micrococcal Nuclease (NEB) was used for digestion of each sample. DNA was purified with phenol: chloroform extraction, ethanol precipitation and subsequently separated on 2% agarose gels. Bands corresponding to ~150 bp were gel isolated, purified, and used for qPCR measurements. Relative nucleosome occupancy was represented as fraction of undigested chromatin DNA and plotted against the *ICE1, MYB15* and *CBF1* genes position with respect to the TSS (transcription start site) for each primer pair where the position denotes the center of each 70–110 bp amplicon. For each experiment three biological and three technical replicates were used.

### 4.6. Microscopic Analyses

Immature seeds were cleared in Hoyer’s solution (30 mL of water, 100 g of chloral hydrate, 7.5 g of Arabic gum, and 5 mL of glycerin) on a glass slide and examined with a compound microscope equipped with Nomarski optics. For scanning electron microscopy (SEM) the plant material was dried in liquid carbon dioxide and mounted on stubs using double sided adhesive and conductive tabs. Next, plant material was coated with gold and platin before imaging with a Zeiss Supra 40VP SEM (Carl Zeiss NTS, Oberkochen, Germany). For each experiment at least three biological replicates were used.

### 4.7. Freezing Tolerance Assay

To determine the freezing tolerance of *swi3c* plants, the electrolyte leakage and survival rate measurements were used. Electrolyte leakage assay was performed according to the methods described by Guo et al., (2002) [58] and Zhao et al., (2016) [34]. Briefly, rosette leaves from 3 weeks old plants grown on 0.5 Murashige and Skoog medium (Sigma Aldrich) containing 0.5% sucrose and 0.8% agar under long day condition (LD) (16h day/8h night) were excised and placed in Eppendorf tubes containing 100 μl deionized water. To each tube a similar ice cube was added and immediately transferred to a freezing bath cooled to 0°C. When all the tubes were placed in freezing bath the program started according to conditions 1°C decrements every 30 min until reached −8°C. The tubes were removed from freezing bath when samples reached desired temperature. Solutions with leaves were then transferred to a falcon tube containing 20 mL of deionized water. Then tubes were incubated overnight and electroconductivity was measured. After measurements, the tubes were incubated in −80°C overnight and the next day electroconductivity was measured again. The electrolyte leakage was calculated as a percentage ratio of conductivity before and after −80°C incubation. Three biological and technical replicates were used.

The survival freezing assay was performed by incubation of 3 weeks old plant grown on MS medium in freezing incubator by applying following temperature program: start temperature was set at 4°C and then dropped to 0°C by 30 min and then temperature decreased by 1°C every 1 h until reached −8°C. After treatment plants were recovered at control condition for 7 days when survival rate was counted. Three biological and technical replicates were done.

### 4.8. Bioinformatic Analysis of Cis-Regulatory Elements

Bioinformatic analysis of *cis*-regulatory elements was performed using the Multiple EM for Motif Elicitation (MEME) [25] and followed by Gene Ontology for Motifs (GOMo) [59]. MEME analysis was performed with default settings using minw = 4 and maxw = 50 parameters. GOMo analysis was done with default settings.

To get the more comprehensive picture of *cis*-regulatory elements the additional bioinformatic analysis of *cis*-regulatory elements was performed using the Arabidopsis Gene Regulatory Information Server (AGRIS; http://arabidopsis.med.ohio-state.edu/) [60,61,62].

### 4.9. Statistical Analyses

The method used in this paper for statistical analyses was unpaired Student’s T-test.

## Figures and Tables

**Table 1:** The effect of decreased growth temperature on *swi3a* and *swi3b* embryo development.

		Number of Analyzed Seeds	Embryo Lethality [%]	Gametophyte Lethality [%]
**14 °C**	WT	265	0	0
	*swi3a/+*	350	6.29	0
	*swi3b/+*	502	0.80	11.95
22 °C	WT	263	0	0
	*swi3a/+*	310	24.84	0
	*swi3b/+*	244	14.75	34.43

## Supplementary Materials

Supplementary materials can be found at https://www.mdpi.com/1422-0067/21/3/762/s1.

## References

[1] Sarnowska E., Gratkowska D.M., Sacharowski S.P., Cwiek P., Tohge T., Fernie A.R., Siedlecki J.A., Koncz C., Sarnowski T.J. (2016). The Role of SWI/SNF Chromatin Remodeling Complexes in Hormone Crosstalk. Trends Plant. Sci..

[2] Phelan M.L., Sif S., Narlikar G.J., Kingston R.E. (1999). Reconstitution of a core chromatin remodeling complex from SWI/SNF subunits. Mol. Cell.

[3] Farrona S. (2004). The Arabidopsis thaliana SNF2 homolog AtBRM controls shoot development and flowering. Development.

[4] Mlynárová L., Nap J.P., Bisseling T. (2007). The SWI/SNF chromatin-remodeling gene AtCHR12 mediates temporary growth arrest in Arabidopsis thaliana upon perceiving environmental stress. Plant. J..

[5] Sang Y., Silva-Ortega C.O., Wu S., Yamaguchi N., Wu M.F., Pfluger J., Gillmor C.S., Gallagher K.L., Wagner D. (2012). Mutations in two non-canonical Arabidopsis SWI2/SNF2 chromatin remodeling ATPases cause embryogenesis and stem cell maintenance defects. Plant. J..

[6] Wagner D., Meyerowitz E.M. (2002). SPLAYED, a novel SWI/SNF ATPase homolog, controls reproductive development in Arabidopsis. Curr. Biol..

[7] Brzeski J., Podstolski W., Olczak K., Jerzmanowski A. (1999). Identification and analysis of the Arabidopsis thaliana BSH gene, a member of the SNF5 gene family. Nucleic Acids Res..

[8] Sarnowski T.J., Ríos G., Jásik J., Swiezewski S., Kaczanowski S., Li Y., Kwiatkowska A., Pawlikowska K., Koźbiał M., Koźbiał P. (2005). SWI3 subunits of putative SWI/SNF chromatin-remodeling complexes play distinct roles during Arabidopsis development. Plant. Cell.

[9] Bezhani S., Winter C., Hershman S., Wagner J.D., Kennedy J.F., Kwon C.S., Pfluger J., Su Y., Wagner D. (2007). Unique, Shared, and Redundant Roles for the Arabidopsis SWI/SNF Chromatin Remodeling ATPases BRAHMA and SPLAYED. Plant. Cell.

[10] Vercruyssen L., Verkest A., Gonzalez N., Heyndrickx K.S., Eeckhout D., Han S.-K., Jegu T., Archacki R., Van Leene J., Andriankaja M. (2014). ANGUSTIFOLIA3 Binds to SWI/SNF Chromatin Remodeling Complexes to Regulate Transcription during Arabidopsis Leaf Development. Plant. Cell.

[11] Sacharowski S.P., Gratkowska D.M., Sarnowska E.A., Kondrak P., Jancewicz I., Porri A., Bucior E., Rolicka A.T., Franzen R., Kowalczyk J. (2015). SWP73 subunits of arabidopsis SWI/SNF chromatin remodeling complexes play distinct roles in leaf and flower development. Plant. Cell.

[12] Sarnowska E.A., Rolicka A.T., Bucior E., Cwiek P., Tohge T., Fernie A.R., Jikumaru Y., Kamiya Y., Franzen R., Schmelzer E. (2013). DELLA-Interacting SWI3C Core Subunit of Switch/Sucrose Nonfermenting Chromatin Remodeling Complex Modulates Gibberellin Responses and Hormonal Cross Talk in Arabidopsis. Plant. Physiol..

[13] Cairns B.R., Levinson R.S., Yamamoto K.R., Kornberg R.D. (1996). Essential role of Swp73p in the function of yeast SWI/SNF complex. Genes Dev..

[14] Buszewicz D., Archacki R., Palusiński A., Kotliński M., Fogtman A., Iwanicka-Nowicka R., Sosnowska K., Kuciński J., Pupel P., Olędzki J. (2016). HD2C histone deacetylase and a SWI/SNF chromatin remodelling complex interact and both are involved in mediating the heat stress response in Arabidopsis. Plant. Cell Environ..

[15] Chinnusamy V., Ohta M., Kanrar S., Lee B.H., Hong X., Agarwal M., Zhu J.K. (2003). ICE1: A regulator of cold-induced transcriptome and freezing tolerance in arabidopsis. Genes Dev..

[16] Stockinger E.J., Gilmour S.J., Thomashow M.F. (1997). Arabidopsis thaliana CBF1 encodes an AP2 domain-containing transcriptional activator that binds to the C-repeat/DRE, a cis-acting DNA regulatory element that stimulates transcription in response to low temperature and water deficit. Proc. Natl. Acad. Sci. USA.

[17] Liu Q., Kasuga M., Sakuma Y., Abe H., Miura S., Yamaguchi-Shinozaki K., Shinozaki K. (1998). Two transcription factors, DREB1 and DREB2, with an EREBP/AP2 DNA binding domain separate two cellular signal transduction pathways in drought- and low-temperature-responsive gene expression, respectively, in Arabidopsis. Plant. Cell.

[18] Dong C.-H., Agarwal M., Zhang Y., Xie Q., Zhu J.-K. (2006). The negative regulator of plant cold responses, HOS1, is a RING E3 ligase that mediates the ubiquitination and degradation of ICE1. Proc. Natl. Acad. Sci. USA.

[19] Fowler S., Thomashow M.F. (2002). Arabidopsis Transcriptome Profiling Indicates That Multiple Regulatory Pathways Are Activated during Cold Acclimation in Addition to the CBF Cold Response Pathway. Plant. Cell.

[20] Agarwal M., Hao Y., Kapoor A., Dong C.H., Fujii H., Zheng X., Zhu J.K. (2006). A R2R3 type MYB transcription factor is involved in the cold regulation of CBF genes and in acquired freezing tolerance. J. Biol. Chem..

[21] Fowler S.G., Cook D., Thomashow M.F. (2005). Low temperature induction of Arabidopsis CBF1, 2, and 3 is gated by the circadian clock. Plant. Physiol..

[22] Chinnusamy V., Zhu J., Zhu J.K. (2007). Cold stress regulation of gene expression in plants. Trends Plant. Sci..

[23] Earley K.W., Haag J.R., Pontes O., Opper K., Juehne T., Song K., Pikaard C.S. (2006). Gateway-compatible vectors for plant functional genomics and proteomics. Plant. J..

[24] Boyes D.C., Zayed A.M., Ascenzi R., McCaskill A.J., Hoffman N.E., Davis K.R., Görlach J. (2001). Growth Stage–Based Phenotypic Analysis of Arabidopsis. Plant. Cell.

[25] Bailey T.L., Elkan C. (1994). Fitting a mixture model by expectation maximization to discover motifs in biopolymers. Proc. Int. Conf. Intell. Syst. Mol. Biol..

[26] Stracke R., Werber M., Weisshaar B. (2001). The R2R3-MYB gene family in Arabidopsis thaliana. Curr. Opin. Plant. Biol..

[27] Riechmann J.L., Heard J., Martin G., Reuber L., Jiang C.Z., Keddie J., Adam L., Pineda O., Ratcliffe O.J., Samaha R.R. (2000). Arabidopsis transcription factors: Genome-wide comparative analysis among eukaryotes. Science.

[28] Hudson M.E. (2003). Identification of Promoter Motifs Involved in the Network of Phytochrome A-Regulated Gene Expression by Combined Analysis of Genomic Sequence and Microarray Data. Plant. Physiol..

[29] Wu M.-F., Sang Y., Bezhani S., Yamaguchi N., Han S.-K., Li Z., Su Y., Slewinski T.L., Wagner D. (2012). SWI2/SNF2 chromatin remodeling ATPases overcome polycomb repression and control floral organ identity with the LEAFY and SEPALLATA3 transcription factors. Proc. Natl. Acad. Sci. USA.

[30] Dubos C., Stracke R., Grotewold E., Weisshaar B., Martin C., Lepiniec L. (2010). MYB transcription factors in Arabidopsis. Trends Plant. Sci..

[31] Thomashow M.F. (1999). PLANT COLD ACCLIMATION: Freezing Tolerance Genes and Regulatory Mechanisms. Annu. Rev. Plant. Physiol. Plant. Mol. Biol..

[32] Jia Y., Ding Y., Shi Y., Zhang X., Gong Z., Yang S. (2016). The cbfs triple mutants reveal the essential functions of CBFs in cold acclimation and allow the definition of CBF regulons in Arabidopsis. New Phytol..

[33] Park S., Lee C.M., Doherty C.J., Gilmour S.J., Kim Y., Thomashow M.F. (2015). Regulation of the Arabidopsis CBF regulon by a complex low-temperature regulatory network. Plant. J..

[34] Zhao C., Zhang Z., Xie S., Si T., Li Y., Zhu J.-K. (2016). Mutational Evidence for the Critical Role of CBF Genes in Cold Acclimation in Arabidopsis. Plant. Physiol..

[35] Schlichting C.D., Wund M.A. (2014). Phenotypic plasticity and epigenetic marking: An assessment of evidence for genetic accommodation. Evolution (N. Y)..

[36] Chinnusamy V., Zhu J.K., Sunkar R. (2010). Gene regulation during cold stress acclimation in plants. Methods Mol. Biol..

[37] Siddiqua M., Nassuth A. (2011). Vitis CBF1 and Vitis CBF4 differ in their effect on Arabidopsis abiotic stress tolerance, development and gene expression. Plant. Cell Environ..

[38] Zhang X., Fowler S.G., Cheng H., Lou Y., Rhee S.Y., Stockinger E.J., Thomashow M.F. (2004). Freezing-sensitive tomato has a functional CBF cold response pathway, but a CBF regulon that differs from that of freezing-tolerant Arabidopsis. Plant J..

[39] Hannah M.A. (2006). Natural Genetic Variation of Freezing Tolerance in Arabidopsis. Plant Physiol..

[40] Jaskiewicz M., Conrath U., Peterhälnsel C. (2011). Chromatin modification acts as a memory for systemic acquired resistance in the plant stress response. EMBO Rep..

[41] Kumar S.V., Wigge P.A. (2010). H2A.Z-Containing Nucleosomes Mediate the Thermosensory Response in Arabidopsis. Cell.

[42] Park J., Lim C.J., Shen M., Park H.J., Cha J.-Y., Iniesto E., Rubio V., Mengiste T., Zhu J.-K., Bressan R.A. (2018). Epigenetic switch from repressive to permissive chromatin in response to cold stress. Proc. Natl. Acad. Sci. USA.

[43] Gilmour S.J., Zarka D.G., Stockinger E.J., Salazar M.P., Houghton J.M., Thomashow M.F. (1998). Low temperature regulation of the Arabidopsis CBF family of AP2 transcriptional activators as an early step in cold-induced COR gene expression. Plant J..

[44] Euskirchen G.M., Auerbach R.K., Davidov E., Gianoulis T.A., Zhong G., Rozowsky J., Bhardwaj N., Gerstein M.B., Snyder M. (2011). Diverse roles and interactions of the SWI/SNF chromatin remodeling complex revealed using global approaches. PLoS Genet..

[45] Archacki R., Yatusevich R., Buszewicz D., Krzyczmonik K., Patryn J., Iwanicka-Nowicka R., Biecek P., Wilczynski B., Koblowska M., Jerzmanowski A. (2016). Arabidopsis SWI/SNF chromatin remodeling complex binds both promoters and terminators to regulate gene expression. Nucleic Acids Res..

[46] Jordán-Pla A., Yu S., Waldholm J., Källman T., Östlund Farrants A.K., Visa N. (2018). SWI/SNF regulates half of its targets without the need for ATP-driven nucleosome remodeling by Brahma. BMC Genomics.

[47] Lake R.J., Boetefuer E.L., Tsai P.F., Jeong J., Choi I., Won K.J., Fan H.Y. (2014). The Sequence-Specific Transcription Factor c-Jun Targets Cockayne Syndrome Protein B to Regulate Transcription and Chromatin Structure. PLoS Genet..

[48] Zhang D., Li Y., Zhang X., Zha P., Lin R. (2017). The SWI2/SNF2 Chromatin-Remodeling ATPase BRAHMA Regulates Chlorophyll Biosynthesis in Arabidopsis. Mol. Plant..

[49] Wei D., Liu M., Chen H., Zheng Y., Liu Y., Wang X., Yang S., Zhou M., Lin J. (2018). INDUCER OF CBF EXPRESSION 1 is a male fertility regulator impacting anther dehydration in Arabidopsis. PLOS Genet..

[50] Alonso J.M., Stepanova A.N., Leisse T.J., Kim C.J., Chen H., Shinn P., Stevenson D.K., Zimmerman J., Barajas P., Cheuk R. (2003). Genome-wide insertional mutagenesis of Arabidopsis thaliana. Science (80-. ).

[51] Koncz C., Schell J. (1986). The promoter of TL-DNA gene 5 controls the tissue-specific expression of chimaeric genes carried by a novel type of Agrobacterium binary vector. Mol. Gen. Genet. MGG.

[52] Davis A.M., Hall A., Millar A.J., Darrah C., Davis S.J. (2009). Protocol: Streamlined sub-protocols for floral-dip transformation and selection of transformants in Arabidopsis thaliana. Plant. Methods.

[53] Schmittgen T.D., Livak K.J. (2008). Analyzing real-time PCR data by the comparative CT method. Nat. Protoc..

[54] Saleh A., Alvarez-Venegas R., Avramova Z. (2008). An efficient chromatin immunoprecipitation (ChIP) protocol for studying histone modifications in Arabidopsis plants. Nat. Protoc..

[55] Pastore J.J., Limpuangthip A., Yamaguchi N., Wu M.F., Sang Y., Han S.K., Malaspina L., Chavdaroff N., Yamaguchi A., Wagner D. (2011). LATE MERISTEM IDENTITY2 acts together with LEAFY to activate APETALA1. Development.

[56] Boyes D.C. (2001). Growth Stage-Based Phenotypic Analysis of Arabidopsis: A Model for High Throughput Functional Genomics in Plants. Plant. Cell.

[57] Zhu Y., Rowley M.J., Böhmdorfer G., Wierzbicki A.T. (2013). A SWI/SNF Chromatin-Remodeling Complex Acts in Noncoding RNA-Mediated Transcriptional Silencing. Mol. Cell.

[58] Guo Y., Xiong L., Ishitani M., Zhu J.-K. (2002). An Arabidopsis mutation in translation elongation factor 2 causes superinduction of CBF/DREB1 transcription factor genes but blocks the induction of their downstream targets under low temperatures. Proc. Natl. Acad. Sci. USA.

[59] Buske F.A., Bodén M., Bauer D.C., Bailey T.L. (2010). Assigning roles to DNA regulatory motifs using comparative genomics. Bioinformatics.

[60] Palaniswamy S.K., James S., Sun H., Lamb R.S., Davuluri R.V., Grotewold E. (2006). AGRIS and AtRegNet. A Platform to Link cis-Regulatory Elements and Transcription Factors into Regulatory Networks. PLANT Physiol..

[61] Davuluri R.V., Sun H., Palaniswamy S.K., Matthews N., Molina C., Kurtz M., Grotewold E. (2003). AGRIS: Arabidopsis Gene Regulatory Information Server, an information resource of Arabidopsis cis-regulatory elements and transcription factors. BMC Bioinformatics.

[62] Yilmaz A., Mejia-Guerra M.K., Kurz K., Liang X., Welch L., Grotewold E. (2011). AGRIS: The arabidopsis gene regulatory information server, an update. Nucleic Acids Res..

